# Teeth loss, teeth brushing and esophageal carcinoma: a systematic review and meta-analysis

**DOI:** 10.1038/srep15203

**Published:** 2015-10-14

**Authors:** Hui Chen, Shuping Nie, Yuhui Zhu, Ming Lu

**Affiliations:** 1 Clinical Epidemiology Unit, Qilu Hospital of Shandong University, Jinan 250012, China; 2 Department of Epidemiology, School of Public Health, Shandong University, Jinan 250012, China

## Abstract

Esophageal carcinoma (EC) is a serious malignancy, and its epidemiologic etiology is not fully explained. We performed this review to investigate the association between teeth loss and teeth brushing and the risk of EC. A systematic search was conducted to identify all relevant studies. The *Q* test and *I*^2^ statistic were used to examine between-study heterogeneity. Pooled odds ratios (ORs) with corresponding 95% confidence intervals (CIs) were considered by fixed or random effects models. Furthermore, we conducted subgroup analyses based on study design, the studies’ geographic regions and case type of origin. Modified Egger linear regression test was used to estimate publication bias. Ten articles were included. Pooled analyses indicated that teeth loss was associated with an increased risk of EC for Asians (OR, 1.52; 95% CI: 1.30, 1.78), and high frequency of teeth brushing was associated with a lower incidence of EC (OR, 0.62; 95%CI: 0.43, 0.89). Subgroup analyses showed consistent results and no publication bias existed. Teeth loss and teeth brushing play potential roles in the progressing of EC. People should take care of their oral health in daily life. And large well-designed researches are needed to fully describe the association between teeth health and EC risk.

Esophageal carcinoma (EC) is the eighth most common incident cancer and the sixth leading cause of cancer death worldwide. EC affects more than 450,000 people worldwide^1^,^2^,^3^, and respect to prognosis and a fatal outcome in the great majority of cases, EC is considered as a serious malignancy^4^. For both of incidence and mortality, the rates of EC were much higher in rural areas than in urban areas, in males than in females^5^. The reviews on the overall age-specific incidence and mortality rates of EC showed that both rates were relatively low before 45 years old, and then gradually increased, reaching peak in the seventh or eighth decades of life^2^,^5^.

Squamous cell carcinoma and adenocarcinoma are the commonly seen forms of EC worldwide. Esophageal squamous cell carcinoma (ESCC) is the predominant form in developing countries, whereas a shift in epidemiology has been seen for some developed countries, where the incidence of esophageal adenocarcinoma (EAC) now exceeds that of squamous-cell types^1^,^6^,^7^.

Numerous epidemiologic investigations and researches have focused on the epidemiological etiology to explain the rapid increase of this lethal cancer^8^,^9^,^10^. Tobacco use, alcohol consumption and mutations of enzymes that metabolizing alcohol have been the primary causes of ESCC, however, alcohol consumption is not considered as a risk factor for EAC^11^,^12^,^13^. For EAC, symptomatic gastro-esophageal reflux disease, Barrett’s esophagus, obesity are considered as major risk factors^7^,^14^,^15^. Therefore, as for teeth loss and the frequency of teeth brushing, the association with EC risk is controversial. Poor oral health has been associated with increased risk of cancer at several sites (i.e. oral cancer, gastric cancer, head and neck cancer, throat cancer etc.)^16^,^17^,^18^,^19^, and other chronic diseases such as cardiovascular disease^20^,^21^ and diabetes^22^ are reported to have an association with poor oral hygiene.

Studies have researched on the association between teeth loss and teeth brushing and the risk of EC, but with inconsistent results^1^,^23^,^24^. Thus, the aims of this study were to carry out a meta-analysis regarding the contributions of teeth brushing and teeth loss to the risk of EC.

## Methods

The Preferred Reporting Items for Systematic reviews and Meta-Analysis (PRISMA) guidelines were followed for the current study^25^.

### Search strategy

Studies that investigated the association between teeth loss and frequency of teeth brushing and EC risk were identified using a search strategy in the following databases: Medline, Embase, Google Scholar, ISI Web of Science, Cochrane Central Register of Controlled Trials to Aug 1st, 2014. Search terms were listed as follows: “oral hygiene” or “oral care” or| “oral health” or “tooth loss” or “teeth loss” or “dental health” or “toothbrushing” or “tooth brushing” or “teeth brushing” or “mouthwash” or “mouthwashes”, “esophageal” or “esophagus” or “oesophagus” or “oesophageal”, “cancer” or “carcinoma” or “tumor” or “neoplasm”. Moreover, we reviewed the reference lists from retrieved articles to search for further relevant studies. When the same data were reported in more than one publication, only the studies with more complete data and more extensive interval of enrollment were included in the study. We followed standard criteria for conducting meta-analyses and reporting the results.

### Eligibility criteria

Each identified study was independently reviewed by two investigators (Chen and Nie) to determine whether an individual study was eligible for inclusion in this meta-analysis. The inclusion criteria are as follows: (1) case-control or cohort study design; (2) exposure of interest was teeth health, including number of teeth loss and the frequency of teeth brushing; (3) outcome of interest was EC; (4) odds ratio (OR) or relative risk (RR) with 95% confidence interval (CI; or data to calculate them) had to be clearly described in the original study.; (5) only articles published in English and studies performed in humans were included. and (6) animal studies, reviews, comments, and editorials were excluded. When there was disagreement between the two investigators about eligibility of the article, it was resolved by consensus with a third reviewer (Zhu).

### Data extraction and quality assessment

A preset data sheet was developed to extract information from the retrieved studies. From each included study, the following data were extracted: first author, publication year, location where the study was performed, characteristics of study population, number of study sample, study results (ORs/RRs and 95% CI ). Both teeth loss and teeth brushing were categorized in 2 levels: the lowest teeth loss group (reference group) and the highest teeth loss group; the lowest frequency teeth brushing group (reference group) and the highest frequency teeth brushing group. Two reviewers extracted all the data independently.

The quality of the included studies was estimated by the 9-star Newcastle-Ottawa Scale and Agency for Healthcare Research and Quality (http://www.ohri.ca/programs/clinical_epidemiology/oxford.asp, maximum score 9 points). This scale assessed the selection of patient, the comparability of group, and the quality of the sampling process.

### Statistical analysis

Pooled measure was calculated as the inverse variance-weighted mean of the logarithm of effects (RRs/HRs/ORs with 95% CI) to assess the strength of association between teeth loss and frequency of teeth brushing and EC. Tests for among-study heterogeneity were performed using the *Q* test and Higgins *I*^2^ statistics^26^. In the presence of substantial heterogeneity (*I*^2^ > 50%), the DerSimonian and Laird random effects model (REM) was adopted; otherwise, we used the fixed effects model (FEM) as the pooling method. The ‘leave one out’ sensitivity analysis was carried out using *I*^2^ > 50% as the criteria to evaluate the key studies with substantial impact on between-study heterogeneity^27^. Publication bias was estimated by Egger’s regression asymmetry test^28^. Data analyses were performed using Stata (version 13.1; Stata Corporation, College Station, TX,USA) software. All reported probabilities (*p*-values) were two-sided, and the values less than 0.05 were considered significant.

## Results

### Study characteristics

**Figure 1:**
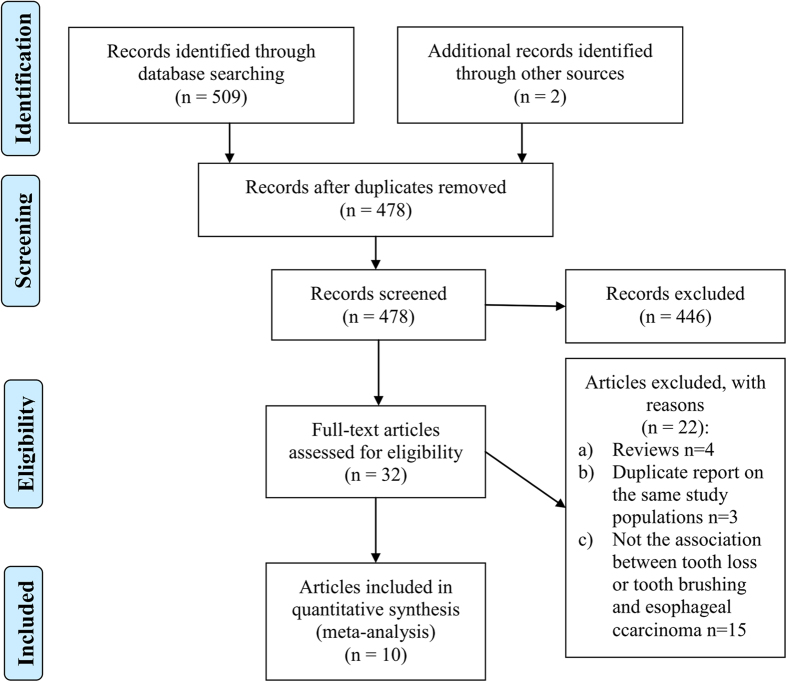
Flow diagram of study selection based on the eligibility criteria.

Figure 1 showed the detailed steps of literature search, and for the 478 potentially relevant articles, ten articles^29^,^30^,^31^,^32^,^33^,^34^,^35^,^36^,^37^,^38^ with twelve studies ultimately met the inclusion criteria for this meta-analysis. These ten included articles were published between 1992 and 2014 and among them six articles^29^,^30^,^31^,^34^,^35^,^36^ with seven studies reported the association between teeth brushing and EC risk, eight articles^29^,^31^,^32^,^33^,^35^,^36^,^37^,^38^ with nine studies reported the association between teeth loss and EC risk. The study design of original articles reported teeth brushing and EC risk were all case-control studies. For articles reported teeth loss and the risk of EC, six were case-control design and three cohort. For the participants of the included articles, six articles included ESCC only, and the remaining articles included EC patients (ECs include ESCCs and EACs. In these studies they just analyze EC as a whole, and did not shown the results of ESCCs and EACs separately.) Most studies provided risk estimates that were adjusted for age (11 studies), sex (9 studies), smoking (9 studies), drinking (9 studies), fruit and vegetable consumption (7); fewer were adjusted for residence (3 studies), BMI (3 studies), education (3 studies). Quality of the included studies was assessed using the Newcastle Ottawa Scale, and all the studies were scored 7 or above out of a possible nine. The details of all the included studies are shown in Table 1 and Table 2.

### Frequency of teeth brushing and EC risk

**Figure 2:**
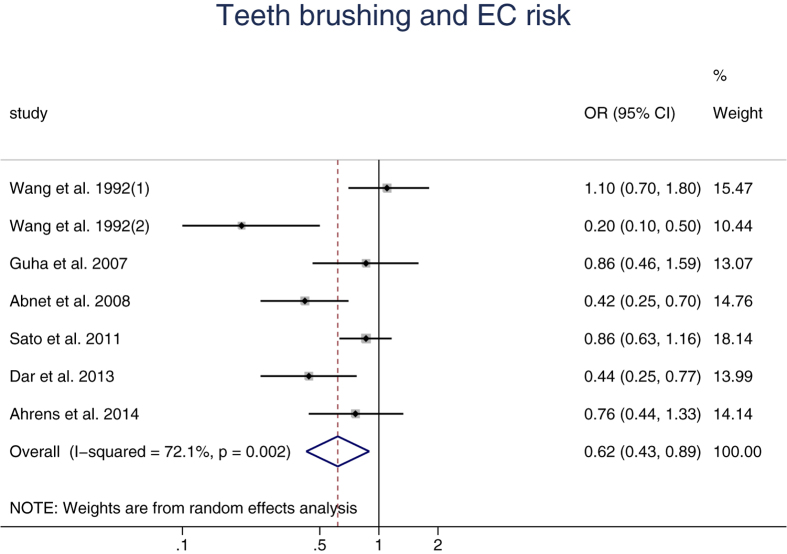
Forest plot for the association between teeth brushing and esophageal carcinoma risk.

The meta-analysis of the association between teeth brushing and EC risk consisted of six articles with seven studies. Five studies in Asia, one in America and one in Europe. The individual estimated ORs and the pooled ORs were presented in Fig. 2. High heterogeneity (*I*^2^ = 72.1%, *p* = 0.002) existed among the studies and the pooled meta-analysis indicated a significant association between teeth brushing and EC risk by REM with an OR = 0.62 (95% CI: 0.43,0.89). Compared with the reference group, people have more frequency of teeth brushing have a lower risk of EC.

Four articles^29^,^31^,^34^,^35^,^36^ with four studies reported the association between teeth brushing and ESCC risk. Pooled results showed a decreased risk of ESCC with people who have more frequency of teeth brushing (pooled results were shown in Table 3).

Subgroup analysis was conducted based on the study’s original design and study location, respectively. Results showed that teeth brushing was associated with the risk of ESCC, and people with high frequency of teeth brushing had a lower incidence of EC in Asia (details were shown in Table 3).

To further explore the potential sources of heterogeneity and the effects of study characteristics on the overall estimates, exploratory meta-regression was performed with study-location (Asia , Europe and America) and source of controls (PB or HB). However, neither of the variables was identified as potential source of between-study heterogeneity. In the sensitivity analysis, no study was found to be a key contributor to between-study heterogeneity.

### Number of teeth loss and EC risk

**Figure 3:**
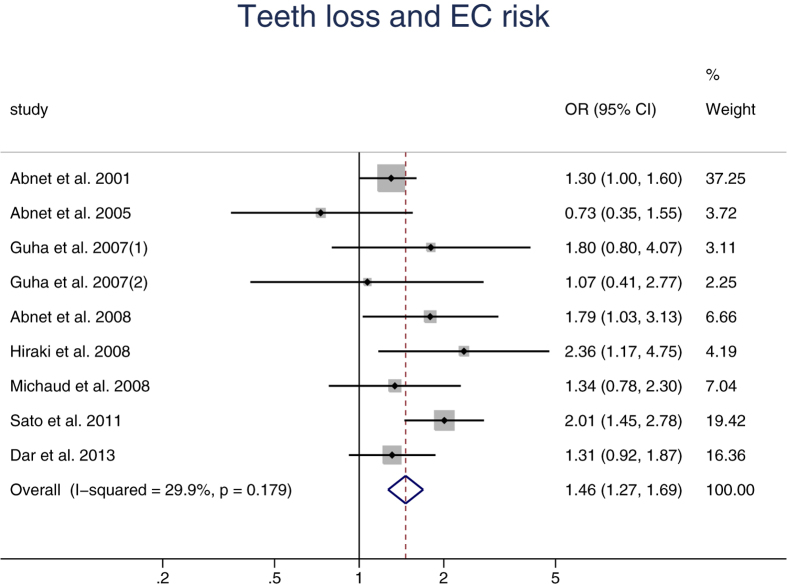
Forest plot for the association between teeth loss and esophageal carcinoma risk.

Eight articles with nine studies regarding the relationship between teeth loss and the risk of EC were included in the meta-analysis. Five studies were conducted in Asia, two in America and two in Europe. The risk estimates for each study and the summary ORs were shown in Fig. 3. No between-study heterogeneity was observed (*I*^2^ = 29.9%, *p* = 0.179) and pooled results showed that teeth loss was related to the occurrence of EC, OR = 1.46 (95% CI: 1.27,1.69). Compared with people who had less teeth loss, people who had more teeth loss had a 46 percent increased incidence of EC.

Subgroup analysis was conducted based on the study’s original design, study-location and case type, respectively. Results showed that teeth loss was associated with the risk of ESCC, and for both cohort studies and case-control studies the results were consistent. People with more teeth loss had a higher incidence of EC in Asia, whereas teeth loss was not significantly associated with EC risk in America and Europe (details were shown in Table 3).

The estimate OR and 95%CI in Sato *et al.* 2011 did not adjusted for potential confounding factors. When excluded this article in the pooled analysis, the results were stable (OR = 1.36 , 95% CI: 1.16, 1.59; *I*^2^ = 0).

### Potential publication bias

**Figure 4:**
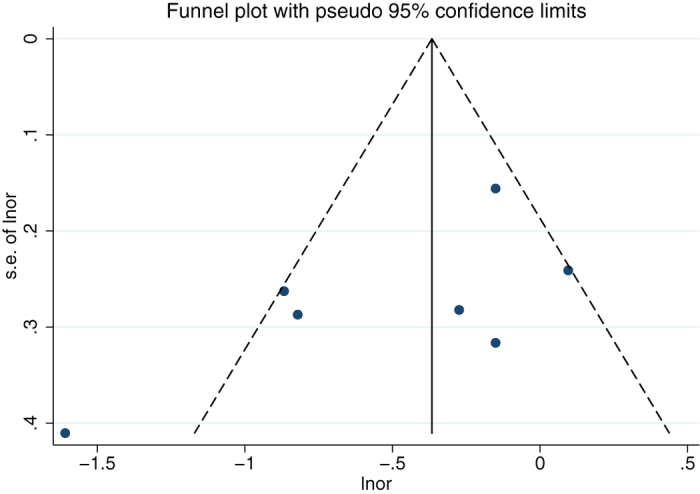
Funnel plot for the association between teeth brushing and esophageal carcinoma.

**Figure 5:**
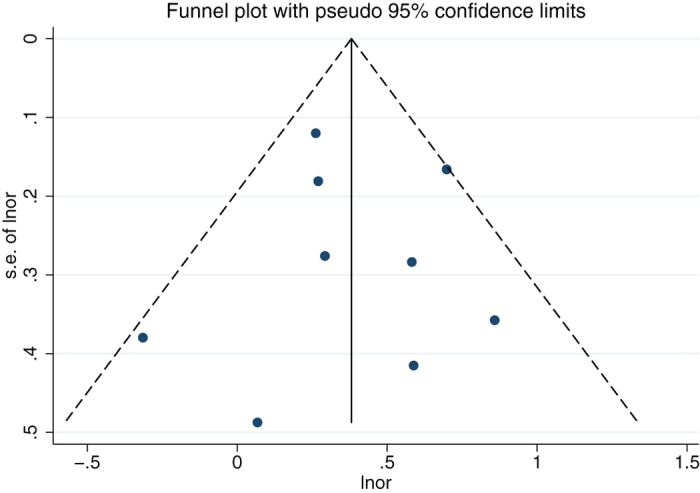
Funnel plot for the association between teeth loss and esophageal carcinoma.

No publication bias was observed in the above-mentioned analyses by the modified Egger linear regression test (with the *p* values 0.132, 0.974, respectively. Table 3). Figures 4 and 5 showed the funnel plots.

## Discussion

The results of this meta-analysis suggested that both teeth brushing and teeth loss were associated the risk of EC. People with higher frequency of teeth brushing had a lower risk of EC. People who had more teeth loss had a higher incidence of EC for Asians, but not for Americans and Europeans. Further subgroup analyses showed consistent results.

To our knowledge, the present meta-analysis is the first one to investigate association between teeth loss, teeth brushing and the risk of EC. The specific mechanisms underlying the association of teeth loss and EC risk are not fully understood. Generally, our results are, in part, consistent with other evidence of increased risk of gastric cancer, head and neck cancer, pancreatic cancer etc.^16^,^17^. For EC, one potential explanation is that teeth loss might alter the dietary pattern to one that increases the risk of disease^39^. Second, we hypothesized that teeth loss would cause individuals to swallow large, poorly chewed boluses of food which might irritate mechanical trauma on the esophagus. Third, teeth loss is associated with an oral flora which may reduce the process of nitrate to nitrite^40^,^41^. This nitrite can then spontaneously react with amines and be converted to carcinogenic nitrosamines, some of which be gastrointestinal organ-specific carcinogens^42^,^43^.

Between-study heterogeneity is common in meta-analyses and characteristics that vary among studies, such as published year, study-location, source of controls, design and quality of original article might act as the sources of between-study heterogeneity^44^,^45^. Our meta-analysis showed significant between-study heterogeneity for teeth brushing and EC risk. Therefore, meta-regression and “leave one out” sensitivity analysis did not find the potential contributors for between-study heterogeneity.

There are limitations in our present meta-analysis. First, our study only included articles published in English, and the number of studies included in this research was limited, which might induce false or unstable results. Second, grouping methods of teeth loss and teeth brushing were varied and complex in the original studies, which made it difficult to regroup them. Therefore, we just calculated data of the high level of teeth loss or teeth brushing compared with the low level without considering the middle groups. Third, for teeth brushing and EC risk, significant between-study heterogeneity existed and we could not find potential contributors, although REM was applied, the pooled results might skewed. Forth, owing to the small number of European and American studies, the selection bias was unavoidable and the association among different regions remained unclear. Last but not least, most of the included studies utilized a case-control design (retrospective study), a design that is more vulnerable to recall bias or changes in exposure related to the disease. The results of this study should be interpreted with caution.

Despite the above disadvantages, the present meta-analysis showed a new aspect on identifying risk factors of EC. No publication bias was observed and subgroup analyses showed consistent results, which indicate that our main findings are robust and not artifact of unpublished negative studies. This meta-analysis suggests that teeth loss significantly increases the risk of EC in Asia, and daily tooth brushing decreases EC risk. And large well-designed researches are needed to fully describe association between oral health and the incidence of EC.

## Additional Information

**How to cite this article**: Chen, H. *et al.* Teeth loss, teeth brushing and esophageal carcinoma: a systematic review and meta-analysis. *Sci. Rep.* **5**, 15203; doi: 10.1038/srep15203 (2015).

## Figures and Tables

**Table 1:** Characteristics of studies on the association between teeth brushing and esophageal carcinoma risk.

First author, year	Design	Location/Setting	Case type	Time periods	Sample size (Case/Control)	Exposure (teeth brushing, times/day)	Risk estimates (OR and 95%CI)	Adjustment factors	Quality score
Wang, 1992	C-C	China, PB	EC	1988–1989	210/203116/189	Yes vs. No	1.1 (0.7, 1.8)^1^ 0.2 (0.1, 0.5)^2^	Age, gender and occupation	7
Guha, 2007	C-C	Latin America, HB	ESCC	1998–2003	91/566	>=2 vs. <=1	0.86 (0.63, 1.16)	Age, sex, education, tabacoo and alcohol consumption	8
Abnet, 2008	C-C	Iran, PB	ESCC	2003–2007	300/571	>=1 vs. 0	0.42 (0.25, 0.70)	Age, sex, ethnicity, smoking and drinking status, hot beverage, fruit and vegetable intake	8
Sato, 2011	C-C	Japan, HB	EC	2001–2005	387/1230	>=2 vs. 1	0.86 (0.63, 1.16)	Age, sex, BMI, occupation, smoking and drinking status, hot beverage, fruit and vegetable intake	7
Dar, 2013	C-C	India, HB	ESCC	2008–2012	703/1664	>=1 vs. 0	0.44 (0.25, 0.77)	Age, ethnicity, residence, education, wealth score, fruit and vegetable intake, smoking and drinking status	7
Ahrens, 2014	C-C	Multi#, HB	ESCC	2002–2005	234/1993	>=3 vs.<1	0.76 (0.44, 1.33)	Age, sex, education, smoking and drinking status, fruit and vegetable intake	8

C-C, case-control; PB, population based; HB, hospital based; EC, esophageal carcinoma; ESCC, esophageal squamous cell carcinoma; BMI, body mass index.

The study wang *et al.* was conducted two locations of China, Yangcheng and Linfen. For “risk estimates”.

^1^represents result in Yangcheng, and.

^2^in Linfen.

^#^Multi, 9 European countries.

**Table 2:** Characteristics of studies on the association between teeth loss and esophageal carcinoma risk.

First author, year	Design	Location/Setting	Case type	Time periods	Sample size (case/control)	Exposure	Risk estimates (OR/RR and 95%CI)	Adjustment factors	Quality score
Abnet, 2001	Cohort	China, PB	ESCC	1986–1991	620/27,715	Any lost teeth vs. no lost teeth	1.3 (1.0, 1.6)	Age, sex, tabacco and alcohol use	8
Abnet, 2005	Cohort	Finland, PB	ESCC	1993–1999	49/28,830	Edentulous vs. 0–10 lost teeth	0.73 (0.35, 1.55)	Age and education	8
Guha, 2007	C-C	Multi*, HB	ESCC	1998–2003	91/566	16–32 lost teeth vs. 0–5 lost teeth	1.80 (0.80, 4.07)^1^	Age, sex, education, tabacoo and alcohol consumption	8
					95/359		1.07 (0.41, 2.77)^2^		
Abnet, 2008	C-C	Iran, PB	ESCC	2003–2007	300/571	Edentulous vs. 0–12 lost teeth	1.79 (1.03, 3.13)	Age, sex, ethnicity, smoking and drinking status, hot beverage, fruit and vegetable intake	8
Hiraki, 2008	C-C	Japan, HB	EC	2001–2005	354/708	Edentulous vs. 0–11 lost teeth	2.36 (1.17, 4.75)	Age, sex, BMI, smoking and drinking status, hot beverage, fruit and vegetable intake, and regular exercise	7
Michaud, 2008	Cohort	USA, PB	EC	1986–2002	131/42,655	16–32 lost teeth vs. 0–7 lost teeth	1.34 (0.78, 2.30)	Age, race, physical activity, BMI, fruit and vegetable intake, smoking and drinking status	9
Sato, 2011	C-C	Japan, HB	EC	2001–2005	387/1230	24–32 lost teeth vs. 0–11 lost teeth	2.01 (1.45, 2.78)	Not adjusted	7
Dar, 2013	C-C	India, HB	ESCC	2008–2012	703/1664	Any lost teeth vs. no lost teeth	1.31 (0.92, 1.87)	Age, ethnicity, residence, education, wealth score, fruit and vegetable intake, smoking and drinking status	7

C-C, case-control; PB, population based; HB, hospital based; ESCC, esophageal squamous cell carcinoma; EC, esophageal carcinoma; BMI, body mass index.

^*^The study Guha *et al.* was conducted in multi locations (Latin America and Central Europe). For “risk estimates”.

^1^represents result in Latin America, and.

^2^in Central Europe.

**Table 3:** Results of overall and subgroup analyses of pooled ORs and 95% CIs.

		No. of included studies	Heterogeneity		Analysis model	OR (95% CI)	*P* for bias
Total and subgroups			*I*^2^(%)	*P* for *Q* test			
Teeth brushing and EC Risk							
All		7	72.1	0.002	REM	0.62 (0.43, 0.89)	0.132
Case type: ESCC		4	38.7	0.180	FEM	0.57 (0.43, 0.76)	
Region: Asia		5	80.8	<0.001	REM	0.55 (0.33, 0.91)	
Teeth loss and EC Risk							
All		9	29.9	0.179	FEM	1.46 (1.27, 1.69)	0.974
Case type: ESCC		6	0	0.501	FEM	1.31 (1.11, 1.56)	
Region	Asia	5	43.3	0.133	FEM	1.52 (1.30, 1.78)	
	America	2	0	0.536	FEM	0.84 (0.47, 1.52)	
	Europe	2	0	0.554	FEM	1.47 (0.94, 2.30)	
Design	Cohort	3	8.2	0.337	FEM	1.25 (1.02, 1.54)	
	Casecontrol	6	0	0.430	FEM	1.69 (1.39, 2.07)	

EC, esophageal carcinoma; ESCC, esophageal squamous cell carcinoma; REM, random effects model; FEM, fixed effects model.
